# Cholesterol‐sensing liver X receptors stimulate Th2‐driven allergic eosinophilic asthma in mice

**DOI:** 10.1002/iid3.118

**Published:** 2016-08-02

**Authors:** Muriel Smet, Lien Van Hoecke, Ans De Beuckelaer, Seppe Vander Beken, Thomas Naessens, Karl Vergote, Monique Willart, Bart N. Lambrecht, Jan‐Åke Gustafsson, Knut R. Steffensen, Johan Grooten

**Affiliations:** ^1^ Department of Biomedical Molecular BiologyGhent UniversityGhentBelgium; ^2^ Medical Biotechnology CenterFlanders Institute for BiotechnologyGhentBelgium; ^3^ Department of Respiratory MedicineGhent University HospitalGhentBelgium; ^4^ Inflammation Research CenterFlanders Institute for BiotechnologyGhentBelgium; ^5^ Department of Biosciences and Nutrition at NovumKarolinska InstitutetStockholmSweden; ^6^ Department of Biology and BiochemistryUniversity of HoustonHoustonTexas; ^7^ Department of Laboratory MedicineKarolinska InstitutetStockholmSweden

**Keywords:** asthma, cholesterol, eosinophilic airway inflammation, liver X receptor

## Abstract

**Introduction:**

Liver X receptors (LXRs) are nuclear receptors that function as cholesterol sensors and regulate cholesterol homeostasis. High cholesterol has been recognized as a risk factor in asthma; however, the mechanism of this linkage is not known.

**Methods:**

To explore the importance of cholesterol homeostasis for asthma, we investigated the contribution of LXR activity in an ovalbumin‐ and a house dust mite‐driven eosinophilic asthma mouse model.

**Results:**

In both models, airway inflammation, airway hyper‐reactivity, and goblet cell hyperplasia were reduced in mice deficient for both LXRα and LXRβ isoforms (LXRα^−/−^β^−/−^) as compared to wild‐type mice. Inversely, treatment with the LXR agonist GW3965 showed increased eosinophilic airway inflammation. LXR activity contributed to airway inflammation through promotion of type 2 cytokine production as LXRα^−/−^β^−/−^ mice showed strongly reduced protein levels of IL‐5 and IL‐13 in the lungs as well as reduced expression of these cytokines by CD4^+^ lung cells and lung‐draining lymph node cells. In line herewith, LXR activation resulted in increased type 2 cytokine production by the lung‐draining lymph node cells.

**Conclusions:**

In conclusion, our study demonstrates that the cholesterol regulator LXR acts as a positive regulator of eosinophilic asthma in mice, contributing to airway inflammation through regulation of type 2 cytokine production.

## Introduction

Asthma is a chronic disease of the airways that presents as recurrent episodes of wheezing, shortness of breath, chest tightness, and coughing. These symptoms are associated with airway inflammation, airway remodeling, and airway hyper‐reactivity (AHR). Asthma is a heterogeneous disease and can be subdivided into endotypes based on clinical characteristics, on pathobiological characteristics, including eosinophilic and neutrophilic inflammation, and on factors that associate with or trigger asthma. Early‐onset allergic asthma is the most frequent type of asthma and is associated with IgE production against identifiable allergic triggers and eosinophilic inflammation driven by Th2 cell populations 1, 2. According to the World Health Organization, 235 million people currently suffer from asthma, most of them children (www.who.int/mediacentre/factsheets/fs307/en/). The prevalence of asthma is highest in developed countries, but the disparity is narrowing due to a rising prevalence in low‐ and middle‐income countries as they adopt a more Western‐type lifestyle 3. One of the main objectives in the strategy of the World Health Organization to prevent and control asthma is to analyze its determinants and monitor trends. Dyslipidemia, characterized by high cholesterol levels in the blood, has gained interest as a risk factor for asthma. This is due to the fact that several epidemiological studies have shown a positive association between dyslipidemia and asthma symptom severity both in children and adults 4, 5, 6, 7, 8, 9, 10. In line herewith, hypercholesterolemia in mice has been shown to result in more severe allergen‐induced eosinophilic airway inflammation as well as in increased AHR and goblet cell hyperplasia 11, 12.

Liver X receptors (LXRs) are a subset of nuclear receptors that regulate whole body and cellular lipid and cholesterol metabolism 13. While the inducible isoform, LXRα, is highly expressed in organs involved in lipid metabolism, such as the liver, adipose tissue, and macrophages, the LXR β‐isoform is expressed ubiquitously at a moderate level. LXRs form heterodimers with the retinoid X receptor and regulate the expression of genes containing LXR‐response elements. LXRs act as cholesterol sensors that upon binding of oxidized cholesterol derivatives promote transcription of genes involved in reverse cholesterol transport, such as the lipid transporters ATP‐binding cassette transporter A1 and G1 (ABCA1/ABCG1), lipid carrier proteins like apolipoprotein E (apoE), and receptors involved in lipid uptake such as scavenger receptor BI (SR‐BI) and low‐density lipoprotein receptor (LDL‐R). Besides cholesterol homeostasis, LXRs also regulate fatty acid metabolism through upregulation of the transcription factor sterol regulatory element‐binding protein 1c (SREBP1c) and several lipogenic enzymes such as stearoyl coenzyme A desaturase‐1 (SCD1). Apart from their role in lipid and cholesterol metabolism, LXRs are increasingly emerging as inflammatory mediators, either repressing or promoting innate and adaptive immune responses in different infectious and inflammatory diseases 14, 15, 16, 17. Owing to their ability to sense increased cholesterol levels and to modulate inflammatory signaling, LXRs pose as a potential regulator at the basis of the positive association between cholesterol and asthma. However, several studies have documented an anti‐inflammatory rather than pro‐inflammatory role for LXR in allergic disorders. LXR activation has been shown to negatively regulate IgE production 18 and to reduce IL‐6 production by IgE‐stimulated murine mast cells 19. Also airway remodeling and AHR appear to be modulated by LXR activity. In a first study, LXR agonist treatment of BALB/c mice resulted in an increase in AHR to inhaled methacholine together with a thickening of the airway smooth muscle mass 20. In contrast, a more recent study showed a decrease in AHR to inhaled methacholine upon LXR activation. This coincided with a reduced airway smooth muscle thickness and reduced goblet cell hyperplasia 21. This discrepancy in the role of LXR in asthma highlights the need for an in‐depth study investigating the contribution of LXR activity to the development of allergic eosinophilic asthma.

In the present study, we investigated the role of LXR in eosinophilic airway inflammation by means of an OVA‐ and a house dust mite (HDM)‐induced asthma mouse model. In both models, we found that eosinophilic airway disease is attenuated in mice deficient for both LXR isoforms. This attenuation was accompanied by a reduced AHR to inhaled methacholine along with a reduced production of type 2 cytokines in the lungs and the lung‐draining lymph nodes (LNs). In addition, we demonstrate that LXR activation by administration of the LXR agonist GW3965 leads to an increase in eosinophilic airway inflammation and type 2 cytokine production by mediastinal LN cells. We conclude that LXR activity positively contributes to eosinophilic airway inflammation as well as to the expression of type 2 cytokines.

## Material and Methods

### Mice

C57BL/6 LXRα^−/−^, LXRβ^−/−^, and LXRα^−/−^β^−/−^ mice 22 were bred in the animal facilities of the Ghent University. Experiments were carried out using age‐ and sex‐matched 8–12‐week‐old mice. Mice were housed in specific pathogen‐free conditions in individually ventilated cages in a controlled day‐night cycle and given food and water ad libitum. All experiments were approved by the animal ethics committee of Ghent University, in accordance with European guidelines.

### OVA/alum mouse asthma model

Mice were sensitized to OVA (grade V) (Sigma, St. Louis, MO) by intraperitoneal (i.p.) injection of 100 μg OVA adsorbed on 1 mg alum (Sigma) in 0.5 mL endotoxin‐free PBS (Lonza, Basel, Switzerland) on days 0 and 7. Starting from day 21, mice were challenged for 30 min with an aerosolized solution of 1% OVA (grade III) (Sigma) in endotoxin‐free PBS for 2–8 consecutive days. Mice that were sensitized with OVA/alum and challenged with PBS served as controls.

### HDM mouse asthma model

Mice were sensitized to HDM by intratracheal (i.t.) instillation of 1 μg HDM extracts (Greer Laboratories, Lenoir, NC) on days 0 and 14. Starting from day 21, mice were challenged intranasally with 10 μg HDM for 4 consecutive days. Mice that were sensitized and challenged with PBS served as controls. In the LXR agonist studies, mice were treated with 20 mg/kg GW3965 (Cayman Chemicals, Ann Arbor, MI) by oral gavage 2 h before intranasal HDM challenge.

### Collection of samples

Mice were euthanized by i.p. injection of Nembutal (Medini NV, Oostkamp, Belgium) 24 h after the indicated allergen challenge. Blood was collected by bleeding of the orbital sinus and captured in an eppendorf tube for serum preparation after overnight clotting at 4°C. Bronchoalveolar lavage (BAL) was performed with 4 × 1 mL HBSS (Life Technologies, Thermo Fisher Scientific, Waltham, MA) supplemented with 0.05 mM EDTA. Supernatant from the first 0.5 mL bronchoalveolar lavage fluid (BALF) was used for cytokine analysis. After BAL, the mediastinal LNs and the lungs were isolated. Depending on the read‐out, lungs were either put in 4% paraformaldehyde for histological analysis, kept in tissue culture medium for CD4^+^ T cell isolation, or snap‐frozen in liquid nitrogen for RNA extraction.

### Flow cytometry

Differential cell counting was performed by flow cytometry. Cells were labeled for the surface markers CD3 (145‐2C11), SiglecF (RP/14), CD11c (HL3), CD4 (RM4‐5), Ly6G (1A8) (BD Biosciences, San Jose, CA), CD8 (53–6.7), CD11b (M1/70) (BD Biosciences), and CCR3 (83101) (R&D Systems, Minneapolis, MN). Measurements were performed on a LSRII flow cytometer (BD Biosciences) and analysis was carried out with BD FACS Diva software. The gating strategy that was used to distinguish the different leukocyte cell populations is shown in Supplementary Figure S1.

### Measurement of AHR

For invasive measurement of dynamic airway elastance, compliance, and resistance, mice were anesthetized with urethane, tracheotomized, and intubated with an 18G catheter. Natural breathing was stopped using d‐tubocurarine, followed by mechanical ventilation with a Flexivent apparatus (SCIREQ, Montreal, Canada). Respiratory frequency was set at 120 breaths/min with a tidal volume of 0.2 mL and a positive‐end expiratory pressure of 2 mL H_2_O was applied. Increasing concentrations of methacholine (0–800 µg/kg) were administered via the jugular vein. Dynamic elastance, compliance, and resistance were recorded after a standardized inhalation maneuver given every 10 sec for 2 min. Baseline resistance was restored before administering the subsequent dose of methacholine.

### Gene expression in total lung cells and lung CD4^+^ cells

To assess relative mRNA expression levels in total lung cells, lungs were first snap‐frozen in liquid nitrogen and then individually crushed using a pre‐cooled mortar. Total RNA was prepared with TRIsure (Bioline, Taunton, MA) according to the manufacturer's instructions. To measure cytokine expression levels in lung CD4^+^ cells, lung tissue was pooled per group and single cell suspensions were prepared by digestion of minced tissue with 1 mg/mL collagenase type IV (Sigma) and 150 U/mL DNaseI (Roche Life Science, Basel, Switzerland) for 30 min at 37°C. The digest was passed through a 70 µm cell strainer, and RBCs were lysed with ACK lysis buffer. The CD4^+^ cell fraction was isolated through magnetic cell sorting with CD4 (L3T4) MicroBeads (Miltenyi Biotec, Bergisch Gladbach, Germany) according to the manufacturer's instructions. Total RNA was prepared with the RNeasy Mini Kit (Qiagen, Hilden, Germany). From the RNA, complementary DNA was prepared with Superscript II (Invitrogen, Thermo Fisher Scientific). Quantitative‐PCR was performed with SYBRGreen mix (Bioline) on a Roche LightCycler 480 system (Applied Biosystems, Thermo Fisher Sceintific). The sequences of the forward and reverse primers (Invitrogen) that were used are as follows: *Il‐5* (5′‐ACATGCACCATTGCCACTAG‐3′ and 5′‐ AATAGAAGTGGGCTTACTAGAG‐3′), *Il‐4* (5′‐CCATGCTTGAAGAAGAACTCTAG TGTT‐3′ and 5′‐GACTCATTCATGGTG CAGCTTATC‐3′), *Il‐13* (5′‐TCAGCCATGAAAT AACTTATTGTTTTGT‐3′ and 5′‐CCTT GAGTGTAACAGGCCATTCT‐3′), *Gata3* (5′‐GG CAGAAAGCAAAATGTTTGCT‐3′ and 5′‐TGAGTCTGAATGGCTTATTCACAAAT‐3′), *Stat6* (5′‐GGGTGTTAATGCTCGAATGTGATA‐3′ and 5′‐CACAATGTCTCTATGTTTCT GTATGTTGAG‐3′), *Abca*1 (5′‐CGCAAGCATATGCCTCAT‐3′ and 5′‐C CCATTACATAACACATGGCT‐3′), *Srebp1c* (5′‐AGGCCATCGACTACATCCG‐3′ and 5′‐TCCATAGAC ACATCTGTGCCTC‐3′), and *Scd1* (5′‐TGGGGCTGCTAATCTCTGGG TGTA‐3′ and 5′‐G GCTTTATCTCTGGGGTGGGTTTG‐3′). Expression data were normalized to the expression levels of housekeeping genes *Tbp* (5′‐TCTACCGTGAATCTTGGCTGTAAA‐3′ and 5′‐TT CTCATGATGACTGCAGCAAA‐3′) and *Rpl13a* (5′‐CCTGCTGCTCTCAAGGTTGTT‐3′ and 5′‐TGGCTGTCACTGCCTGGTACTT‐3′).

### Ex vivo restimulation of mediastinal LN cells

Mediastinal LNs were isolated and pooled per group or kept separate, as indicated. A single cell suspension was obtained by passing the LNs through a 70 μm cell strainer. Cells were seeded in round bottom 96 well plates at 1 × 10^6^ cells per well and restimulated either with PBS or with 15 μg/mL HDM. After 48 h, culture supernatants were collected for luminex analysis (Bio‐Rad, Hercules, CA).

### Cytokine, chemokine, and allergen‐specific IgE measurements

Cytokines and chemokines were captured with luminex beads (Bio‐Rad) and the cytokine levels were measured on a Luminex Bioplex suspension array system (Bio‐Rad) according to the manufacturer's instructions. CCL‐11 levels in the BALF were measured using a mouse CCL‐11/Eotaxin ELISA (R&D Systems). OVA‐specific IgE levels in serum and BALF were determined by ELISA. Briefly, plates were coated with OVA (grade V) (Sigma) and incubated with a twofold serial dilution of the serum or BALF samples. Allergen‐bound IgE antibodies were detected with an HRP‐conjugated rat anti‐mouse IgE detection antibody (Southern Biotech, Birmingham, AL). Plates were developed with TMB substrate (BD OptEIA, BD Biosciences) and measurements were made at 450/655 nm using a microplate reader after stopping the reaction with sulphuric acid. The IgE titer was defined as the dilution factor of the sample at a specific absorbance value in the linear area of the dilution curve.

### Histological analysis

Lungs were fixed with 4% paraformaldehyde, embedded in paraffin, cut into 5 µm sections, and stained with periodic acid–Schiff (PAS) (Sigma) to identify mucus‐containing cells. The numerical scores for the abundance of PAS‐positive mucus‐containing cells in each bronchus were determined as follows: 0, less than 5% PAS‐positive cells; 1, 5–25%; 2, 25–50%; 3, 50–75%; 4, >75% as described by Tanaka et al. 23.

### Statistics

For all experiments apart from AHR studies, data are shown as mean ± SD. Statistical analysis between two groups was performed using a Mann–Whitney *U* test and between multiple groups using a Kruskal–Wallis test followed by a post hoc Dunn's test. Significant *P*‐values were ranked as *P* < 0.05 (*), *P* < 0.01 (**), and *P* < 0.001 (***).

For AHR studies, the dynamic airway elastance, compliance, and resistance values were ln‐transformed and analyzed as repeated measurements using the residual maximum likelihood (REML) as implemented in Genstat v17 24. A linear mixed model was fitted to the data, with methacholine dosages set as not‐equally spaced. The antedependence correlation structure of order 1 or 2 was selected as best model fit for each of the three data sets based on a likelihood ratio test (LRT) statistic and the Aikake Information Coefficient (AIC). Significance of the fixed main and interaction effects was assessed by an F‐test. Significances of contrasts between particular genotype and methacholine dose combinations were assessed by an F‐test. Data are shown as mean ± SE. Significant *P*‐values were ranked as *P* < 0.05 (*), *P* < 0.01 (**), and *P* < 0.001 (***).

## Results

### LXR is a positive regulator of OVA‐induced eosinophilic airway disease

**Figure 1:**
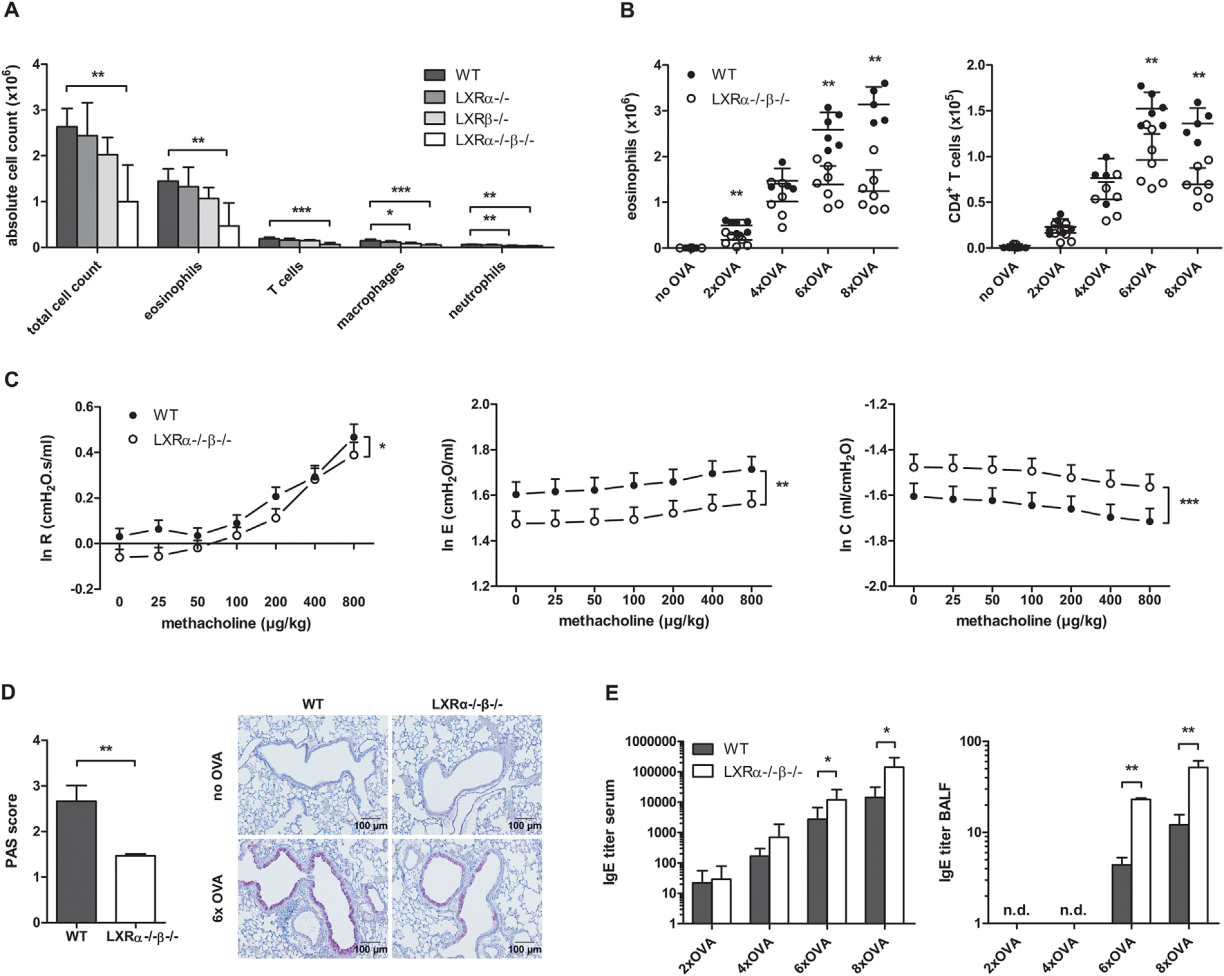
LXR‐deficient mice display reduced airway inflammation, airway hyper‐reactivity (AHR) and goblet cell hyperplasia in OVA‐induced asthma as compared to wild‐type (WT) mice. WT, LXRα^−/−^, LXRβ^−/−^, and LXRα^−/−^β^−/−^ mice were sensitized against OVA and subsequently challenged with aerosolized OVA. (A) Differential cell counts in the bronchoalveolar lavage (BAL) after exposure to six OVA aerosols measured by flow cytometry. (B) Eosinophils and CD4^+^ T cells in the BAL assessed by flow cytometry after 2–8 consecutive OVA aerosols. (C) Dynamic airway resistance [R], elastance [E], and compliance [C] was determined in mice exposed to increasing doses of methacholine after exposure to six OVA aerosols. (D) Mucus production evaluated by PAS staining of lung paraffin sections of mice exposed to six OVA aerosols. (E) OVA‐specific IgE titer in serum and bronchoalveolar lavage fluid measured by ELISA. (A) A Kruskal–Wallis test followed by a post hoc Dunn's test was used. (B, D, and E) Statistical analysis between two groups was performed using a Mann–Whitney *U* test. Data are shown as mean ± SD, *n* = 5 to 7 mice per group. The results show one representative experiment out of at least two independent experiments. (C) The repeated measures in dynamic AHR of three independent experiments were analyzed by residual maximum likelihood. Data are shown as mean ± SE, *n* = 5–7 mice per group. Significant *P*‐values were ranked as *P* < 0.05 (*), *P* < 0.01 (**), and *P* < 0.001 (***).

To investigate the potential role of LXRs in allergic eosinophilic asthma, we examined LXR‐deficient mice in an OVA‐induced eosinophilic asthma mouse model. Mice were first sensitized against OVA by i.p. injection together with aluminium hydroxide (alum) and then exposed to nebulized OVA for 6 consecutive days, which resulted in inflammatory cell infiltration in the lungs. Mice deficient for both LXR isoforms (LXRα^−/−^β^−/−^) showed a decrease in overall airway inflammation, with a twofold reduction in eosinophil numbers in the BAL as compared to wild‐type (WT) mice (Fig. 1A). This reduction in eosinophilic inflammation was associated with the loss of both the LXR α‐ and β‐isoforms since eosinophil numbers in the lungs of LXRα^−/−^ and LXRβ^−/−^ mice were either identical or only slightly decreased as compared to WT mice (Fig. 1A). Because loss of a single isoform was functionally compensated by the other, all following experiments using this model were performed on LXRα^−/−^β^−/−^ mice.

The reduced airway eosinophilia in LXR‐deficient mice was apparent already early during the allergic inflammatory response, after two consecutive aerosol exposures, and persisted at all time‐points assessed (Fig. 1B). In contrast to the diminished eosinophil numbers early in the inflammatory response, CD4^+^ T cell numbers in the BAL of LXRα^−/−^β^−/−^ mice showed reduced numbers only after six OVA aerosols (Fig. 1B).

Next, we assessed AHR of OVA challenged WT and LXRα^−/−^β^−/−^ mice by measuring dynamic resistance, elastance, and compliance of the airways to methacholine 24 h after the last of six daily OVA aerosols. The airway resistance seen in OVA‐challenged LXR‐deficient mice was significantly lower as that seen in OVA‐challenged WT mice, indicative of reduced AHR upon loss of LXR activity (Fig. 1C). In accordance with reduced airway resistance, OVA‐challenged LXRα^−/−^β^−/−^ mice also displayed a reduction in airway elastance and an increase in airway compliance, indicative of a reduced stiffness of the lungs (Fig. 1C). Another main characteristic of asthma is airway narrowing due to excessive mucus production, resulting from goblet cell hyperplasia. In accordance with reduced airway inflammation and reduced AHR, also the number of mucus‐producing goblet cells was diminished in OVA challenged LXR‐deficient mice in comparison to WT mice, as determined by lung histologic PAS staining (Fig. 1D).

Remarkably, when assessing peripheral and local OVA‐specific IgE antibodies, an opposite trend toward increased reactivity was observed. As shown in Figure 1E, LXRα^−/−^β^−/−^ mice showed increased serum levels of OVA‐specific IgE compared to WT mice, reaching statistical significance at six and eight OVA challenges. Local IgE levels in the BALF were lower as compared to those in the serum and were detectable only after six and eight OVA challenges. Similarly to serum, at these time points, increased IgE levels were observed for LXRα^−/−^β^−/−^ mice in comparison to WT mice (Fig. 1E).

### LXR is a positive regulator of HDM‐induced eosinophilic airway disease

**Figure 2:**
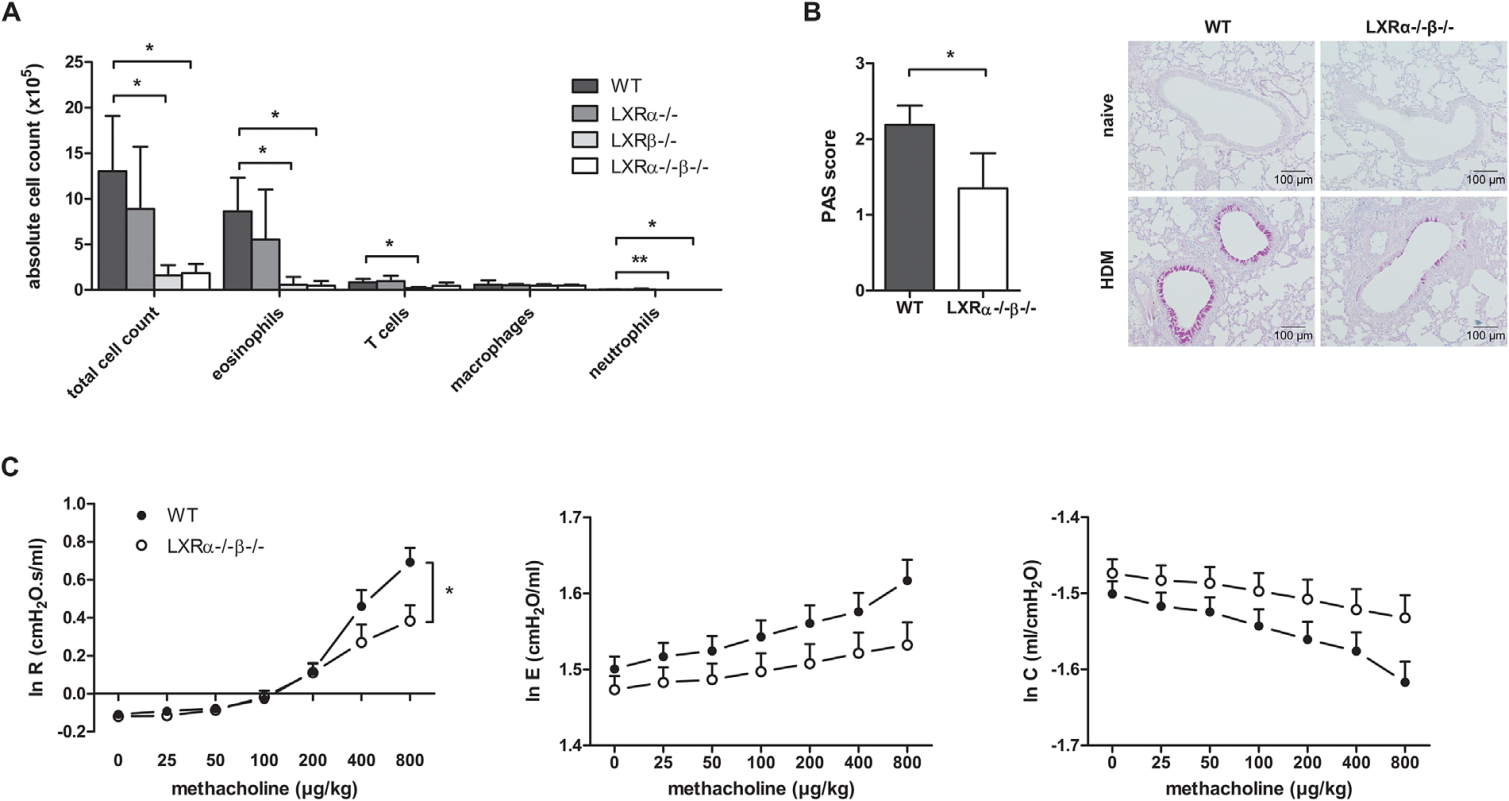
LXR‐deficient mice display reduced airway inflammation, goblet cell hyperplasia, and airway hyper‐reactivity (AHR) in house dust mite (HDM)‐induced asthma as compared to wild‐type (WT) mice. WT, LXRα^−/−^, LXRβ^−/−^, and LXRα^−/−^β^−/−^ mice were sensitized against HDM and subsequently challenged intranasally with HDM for 4 consecutive days. (A) Differential cell counts in the bronchoalveolar lavage measured by flow cytometry. (B) Mucus production evaluated by PAS staining of lung paraffin sections. (C) Dynamic airway resistance [R], elastance [E], and compliance [C] was measured in mice exposed to increasing doses of methacholine after HDM challenge. (A) A Kruskal–Wallis test followed by a post‐hoc Dunn's test was used. (B) Statistical analysis between two groups was performed using a Mann–Whitney *U* test. Data are shown as mean ± SD, *n* = 5–7 mice per group. The results show one representative experiment out of at least two independent experiments. (C) The repeated measures in dynamic AHR of one independent experiment were analyzed by residual maximum likelihood. Data are shown as mean ± SE, *n* = 5–7 mice per group. Significant *P*‐values were ranked as *P* < 0.05 (*) and *P* < 0.01 (**).

In a second approach, we investigated the role of LXR activity in a mouse asthma model driven by the natural allergen HDM 25. In this model, mice were first sensitized to the allergen by i.t. instillation and subsequently challenged four times with HDM administered by the intranasal route. Analysis of the airway inflammatory response revealed an even stronger LXR dependency as compared to the OVA‐driven mouse model. A sevenfold decrease in eosinophil numbers was observed in the BAL of mice deficient for either the LXR β‐isoform alone (LXRβ^−/−^) or for both LXR isoforms (LXRα^−/−^β^−/−^) as compared to WT mice (Fig. 2A), indicating a dependency on the LXR β‐isoform. Aside from a reduction in airway inflammation, LXRα^−/−^β^−/−^ mice also displayed a reduced number of mucus‐producing goblet cells in the airways (Fig. 2B) and reduced AHR (Fig. 2C). LXR‐deficient mice challenged with HDM displayed a significantly lower airway resistance upon exposure to methacholine together with a trend toward a decrease in airway elastance and an increase in airway compliance (Fig. 2C). Finally, serum levels of HDM‐specific IgE were also measured but were too low for reliable titer determination (data not shown).

### LXR deficiency is associated with reduced type 2 cytokine production in allergen‐challenged lungs

**Figure 3:**
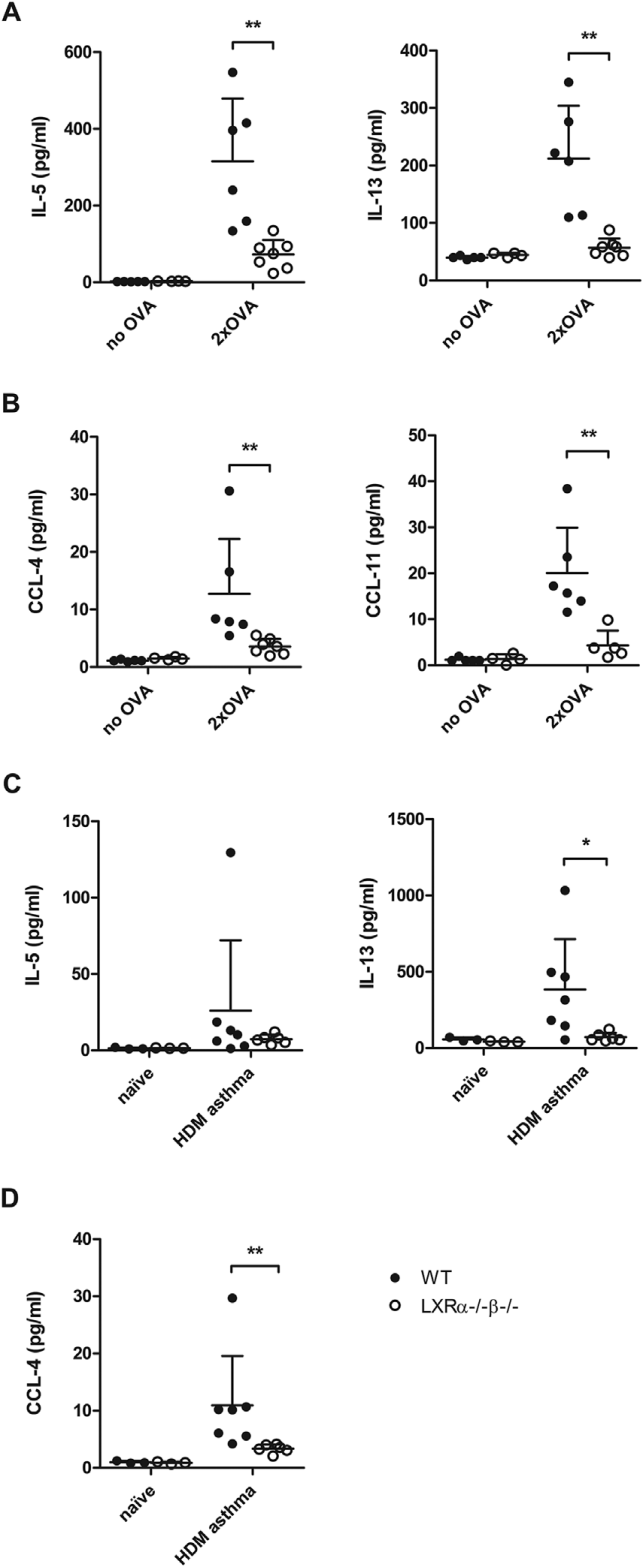
LXR‐deficient mice show decreased type 2 cytokine and chemokine levels in the lungs as compared to wild‐type (WT) mice in OVA‐ and house dust mite (HDM)‐induced asthma. (A) Cytokine and (B) chemokine levels in the bronchoalveolar lavage fluid (BALF) of WT and LXRα^−/−^β^−/−^ mice after 2 consecutive OVA aerosols. (C) Cytokine and (D) chemokine levels in the BALF of WT and LXRα^−/−^β^−/−^ mice after four intranasal HDM challenges. Cytokine and chemokine levels were determined by luminex analysis or ELISA. Data are shown as mean ± SD, *n* = 5–7 mice per group. The results show one representative experiment out of two independent experiments. Statistical analysis between two groups was performed using a Mann–Whitney *U* test. Significant *P*‐values were ranked as *P* < 0.05 (*) and *P* < 0.01 (**).

In order to verify to what extent LXR deficiency affected local production of type 2 cytokines early in the OVA‐driven inflammatory response, we quantified cytokine and chemokine levels in the BALF after two OVA aerosols. Protein levels of the distinctive type 2 cytokines, IL‐5, and IL‐13, but not of IL‐4, were readily detectible in the BALF of OVA‐challenged WT mice (Fig. 3A). Strikingly, in LXRα^−/−^β^−/−^ mice, a pronounced drop in IL‐5 and IL‐13 protein levels was observed, showing protein levels close to those in unchallenged mice. Next to a decreased type 2 cytokine production in the BALF, LXRα^−/−^β^−/−^ mice also showed reduced levels of CCL‐4 (RANTES) and CCL‐11 (eotaxin‐1), chemokines involved in eosinophil recruitment to the asthmatic lung (Fig. 3B).

Also for the HDM‐driven asthma model, we assessed the effect of LXR deficiency on the production of type 2 cytokines and eosinophil‐recruiting chemokines in the lungs. After four consecutive daily HDM challenges, we observed slightly reduced IL‐5 and strongly reduced IL‐13 protein levels in the BALF of LXRα^−/−^β^−/−^ mice as compared to WT mice (Fig. 3C). Likewise, LXR‐deficient mice displayed strongly diminished CCL‐4 levels in the BALF (Fig. 3D). Protein levels of IL‐4 and CCL‐11 were not detectable.

### Loss of LXR activity is associated with reduced type 2 cytokine expression in allergen‐challenged lungs and lung‐draining lymph nodes

**Figure 4:**
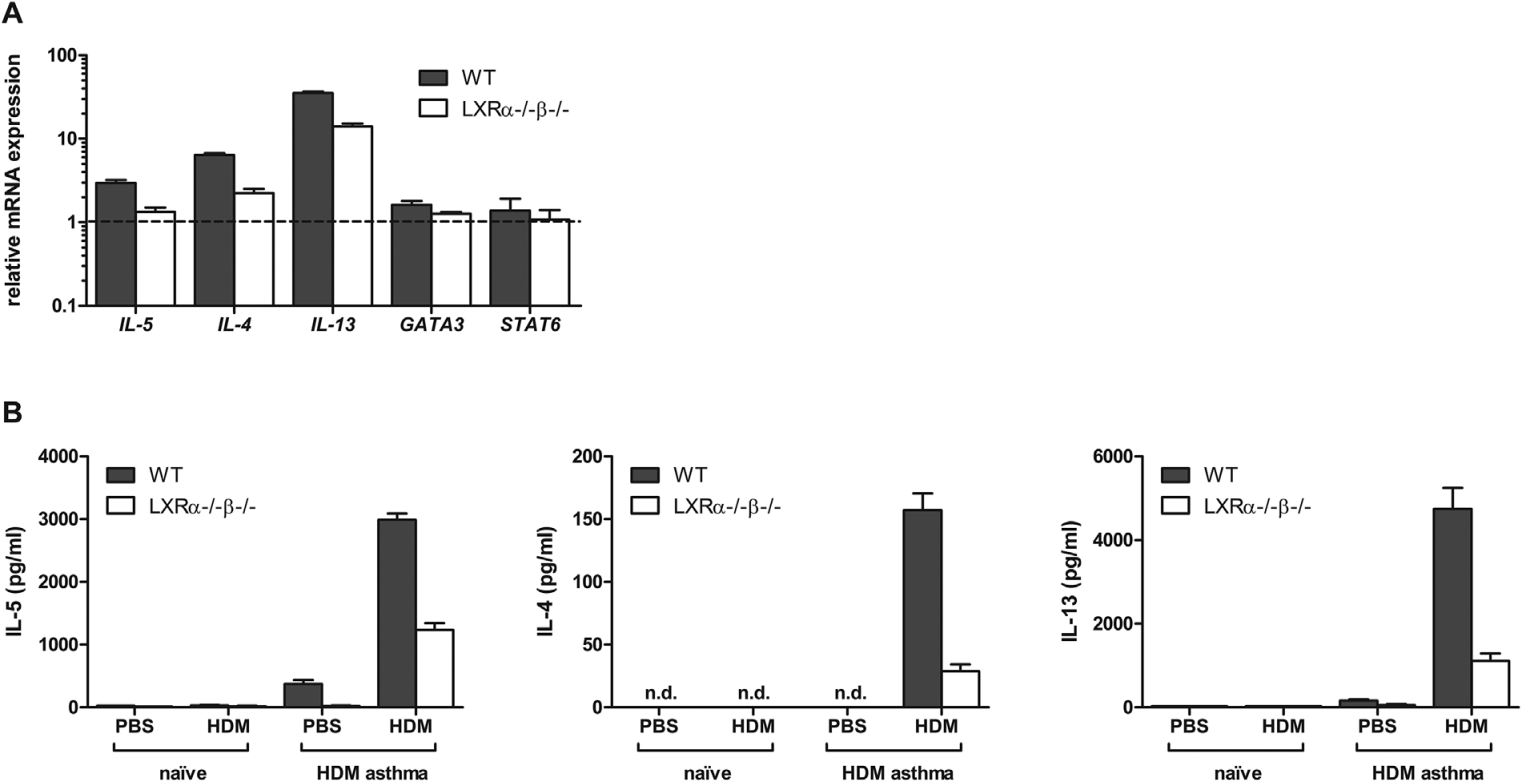
LXR‐deficient mice show decreased Th2 cytokine expression in the lungs and lung‐draining lymph nodes as compared to wild‐type (WT) mice in OVA‐induced and house dust mite (HDM)‐induced asthma. (A) mRNA expression levels of Th2‐associated cytokines (*Il‐5*, *Il‐13*, *Il‐4*) and transcription factors (*Gata3* and *Stat6*) in the CD4^+^ cells of the lungs of WT and LXRα^−/−^β^−/−^ mice after two consecutive OVA aerosols. Lung cells were pooled per group before CD4^+^ enrichment and relative mRNA expression levels were determined in triplicate by qPCR. (B) Cytokine production by mediastinal LN cells of WT and LXRα^−/−^β^−/−^ mice after four intranasal HDM challenges. Mediastinal LN cells were pooled per group and restimulated in quadruplicate with HDM for 48 h. Cytokine levels were measured by luminex analysis. Data are shown as mean ± SD, *n* = 3–7 mice per group. The results show one representative experiment out of two independent experiments.

In addition to reduced type 2 cytokine protein levels in OVA‐driven asthma, diminished mRNA expression levels of *Il‐5*, *Il‐13*, and *Il‐4* were observed in CD4‐enriched lung cells from LXRα^−/−^β^−/−^ mice (Fig. 4A). In contrast, the mRNA levels of the Th2‐regulating transcription factors *Gata3* and *Stat6* were unaltered between WT‐ and LXR‐deficient CD4^+^ lung cells (Fig. 4A), thus indicating that the nature of the Th2 cell response was not affected by LXR deficiency. The lack of mRNA expression of Th1‐ and Th17‐associated cytokines and transcription factors (data not shown) further corroborated the unaltered Th2 nature of the allergic response in LXRα^−/−^β^−/−^ mice.

In the HDM‐driven asthma model, we found that the lung‐draining mediastinal LN cells of HDM‐sensitized and challenged LXRα^−/−^β^−/−^ mice produced less type 2 cytokines upon ex vivo HDM restimulation as compared to LN cells from WT mice (Fig. 4B). In addition, we did not observe a bias toward the Th17 effector cytokine IL‐17 or Th1 effector cytokine IFNγ (data not shown), thus revealing that also in this model the nature of the Th2 response is not changed.

### Treatment with a synthetic LXR agonist increases HDM‐induced eosinophilic airway inflammation

**Figure 5:**
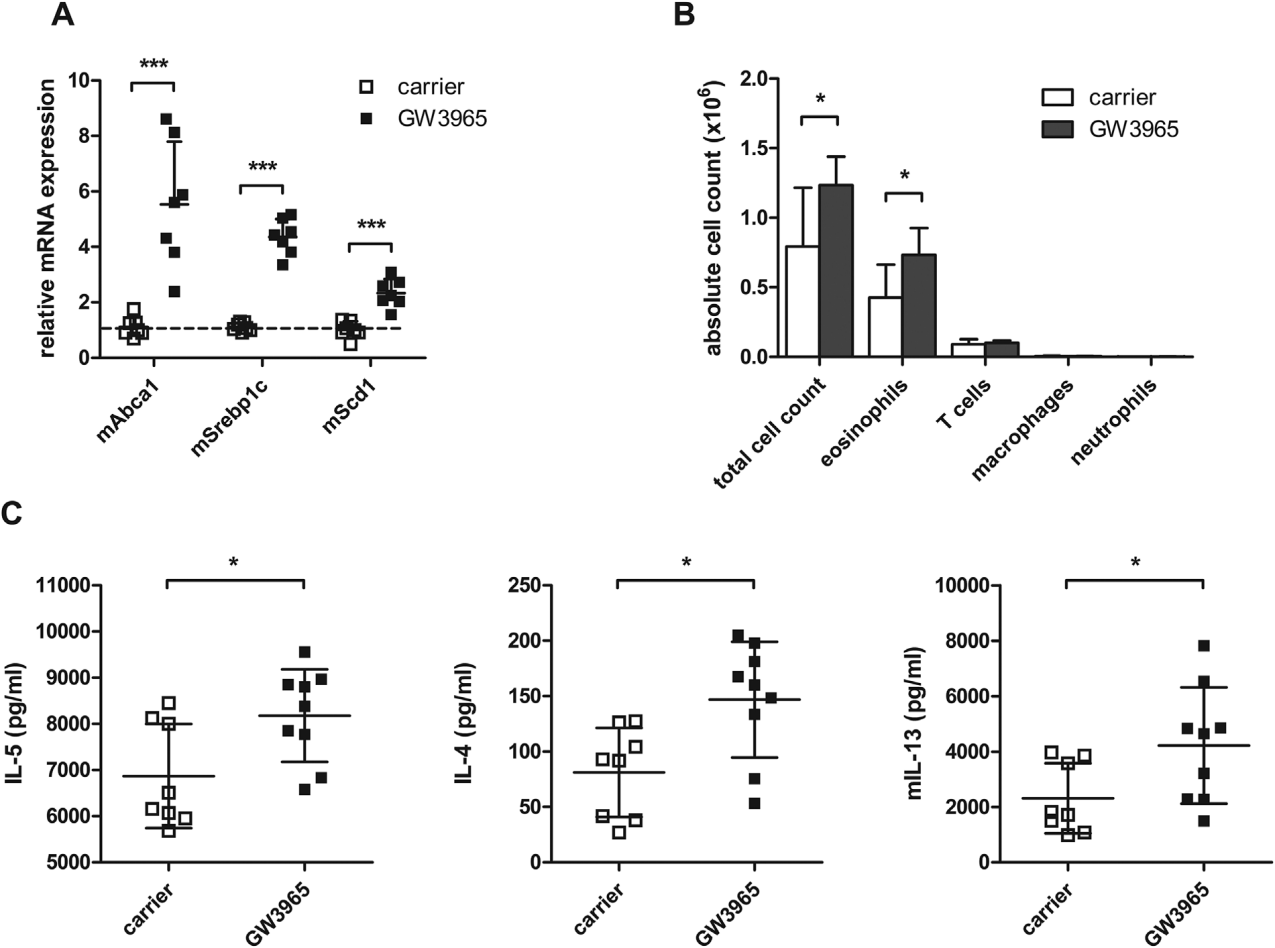
LXR agonist treatment increases eosinophilic airway inflammation and type 2 cytokine production in house dust mite (HDM)‐induced asthma. (A) mRNA expression levels of LXR target genes in the lungs 6 h after carrier or GW3965 treatment by oral gavage. (B) Wild‐type mice were sensitized against HDM and subsequently challenged intranasally with HDM for 4 days. Two hours before intranasal HDM challenge, mice were treated with carrier or GW3965 administered by oral gavage. Differential cell counts in the bronchoalveolar lavage were measured by flow cytometry. (C) Mediastinal lymph nodes were isolated and cells were restimulated with HDM for 48 h. Cytokine levels were measured by luminex analysis. Statistical analysis between two groups was performed using a Mann–Whitney *U* test. Data are shown as mean ± SD, *n* = 7–10 mice per group. The results show one representative experiment out of two independent experiments. Significant *P*‐values were ranked as *P* < 0.05 (*), *P *< 0.01 (**), and *P *< 0.001 (***).

Next, we investigated the effect of LXR activation on eosinophilic airway inflammation. Therefore, we treated mice by oral gavage with 20 mg/kg of the highly selective LXR agonist, GW3965. This treatment resulted in a pronounced upregulation of LXR target genes (*Abca1*, *Srebp1c*, and *Scd1*) in the lungs (Fig. 5A), proving that the agonist reached the lungs and was biologically active. Accordingly, we combined LXR agonist treatment with the HDM‐induced asthma model in order to verify the impact of an increased LXR activity on the airway inflammatory response. As shown in Figure 5B, an increased LXR activity prior to HDM allergen exposure significantly increased the strength of the airway inflammation as indicated by the increased total cell numbers and eosinophils present in the BAL of the treated mice. Consistent with a stronger eosinophilic inflammation upon LXR activation, the mediastinal LN cells of the mice produced higher levels of the type 2 cytokines IL‐5, IL‐4, and IL‐13 after ex vivo HDM restimulation (Fig. 5C).

## Discussion

In this study, we addressed the role of the cholesterol‐sensing nuclear receptor LXR in the pathogenesis of eosinophilic allergic asthma. To this end, we employed a mouse asthma model based on sensitization to the model allergen, OVA, in the presence of the Th2‐skewing adjuvant alum followed by OVA aerosol challenges. We showed that in this mouse model, a significantly attenuated airway inflammation is seen in mice that are deficient for both the α‐ and β‐isoform of LXR. This attenuated airway response was partially lost with single LXRα and LXRβ deficiency, indicative of a functional redundancy for both LXR isoforms in this asthma model. A similar observation was made in a mouse asthma model based on sensitization and challenge with HDM, a naturally occurring allergen 25. However, in the HDM‐induced asthma model, the LXR α‐isoform could not compensate for the loss of LXRβ, thus indicating that here the contribution of LXR activity to airway inflammation was driven primarily by the β‐isoform. Although LXR‐deficient mice represent a good tool to investigate the function of LXR in disease pathogenesis, the use of LXR agonists is complementary to these studies and serves as a strategy to increase LXR activity. Here, we showed that an increased LXR activity directly impacted on the airway allergic response. Administration of the highly selective LXR agonist, GW3965, prior to HDM challenge resulted in increased eosinophilic airway inflammation. Both approaches—loss versus gain of LXR activity—corroborate a role for the cholesterol‐sensing nuclear receptor LXR as a positive regulator of airway inflammation in asthma.

Aside from contributing to airway inflammation, we also demonstrated that loss of LXR activity reduced excessive mucus production and AHR, cardinal features of asthma. In both OVA‐induced and HDM‐induced asthma, LXRα^−/−^β^−/−^ mice displayed strongly diminished numbers of mucus‐producing goblet cells and showed a reduced dynamic airway resistance to methacholine. Our findings are in line with a study showing increased AHR to inhaled methacholine in OVA‐challenged BALB/c mice upon oral treatment with the LXR agonist GW3965 20. In contrast, another study in OVA‐challenged BALB/c mice showed decreased AHR to inhaled methacholine upon oral treatment with the LXR agonist T0901317 21. The cause of these different outcomes could be the use of distinct synthetic LXR agonists. Whereas the GW3965 agonist is highly selective for LXR, the T0901317 agonist can also activate other nuclear receptors such as the Farnesoid X Receptor, indicating that the observations in this study might be LXR‐independent. Collectively, our findings identify LXR as a nuclear receptor contributing to eosinophilic airway inflammation and the pathogenesis of allergic asthma.

An important feature of allergic asthma is the production of allergen‐specific IgE antibodies. Applying the OVA/alum‐based model, we here show OVA‐specific IgE levels in serum and BALF to be increased in LXRα^−/−^β^−/−^ mice. This observation is in line with previous reports documenting a reduction in IgE levels upon LXR activation in OVA/alum‐based models 18, 21. Taken together, our data suggest that LXR activity differentially impacts on distinct branches of the allergic response; on the one hand promoting eosinophilic airway inflammation while on the other hand repressing IgE antibody production and atopy.

Eosinophilic inflammation in asthmatic lungs is mainly driven by CD4^+^ Th2 cells through production of the type 2 cytokines IL‐4, IL‐5, and IL‐13. LXR deficiency strongly affected type 2 cytokine production in both mouse models, marked by reduced protein levels of IL‐5 and IL‐13 in the BALF of LXRα^−/−^β^−/−^ mice as compared to WT mice. Analysis of the reactivity and helper profile of CD4‐enriched cells from the lung of OVA challenged LXR‐deficient mice showed lower mRNA levels of Th2 cytokines in spite of unaltered expression of Th2 differentiation markers and this while the nature of the T helper cell response was not affected. A reduction in the number of CD4^+^ T cells in the BAL of LXRα^−/−^β^−/−^ mice was seen only after six challenges with nebulized OVA, whereas decreased type 2 cytokine levels were observed already after two OVA challenges. This difference in kinetics suggests that type 2 cytokine production was affected directly by LXR activity rather than indirectly through a defective expansion of type 2 cytokine‐producing CD4^+^ T cells. These observations therefore point toward a reduced Th2 cell reactivity as basis of the reduced type 2 cytokine levels in the BALF of LXR‐deficient mice. In the HDM‐driven asthma model, lung‐draining mediastinal LN cells restimulated ex vivo with HDM showed reduced type 2 cytokine expression, again in spite of an unaltered expression of Th2 cell differentiation markers. Inversely, mediastinal LN cells showed an increased production of type 2 cytokines upon an increase of LXR activity induced by treatment with the LXR agonist GW3965. These results all point toward LXR exerting a positive regulatory role on eosinophilic inflammation by acting as a transcriptional regulator of type 2 cytokine expression in T cells. In line herewith, a recent study identified the mouse IL‐5 gene as a direct LXR target gene with a putative LXR response element in its promoter region 26. The IL‐5 gene lies in a Th2 cytokine locus that also encompasses the IL‐4 and IL‐13 gene, located on mouse chromosome 11qB1.3 and human chromosome 5q31.1. These type 2 cytokine‐encoding genes have been shown to be regulated in a coordinate manner 27, 28, 29. Further research will be needed to verify to what extent LXR transcription factor activity directly regulates the expression of type 2 cytokine‐encoding genes and hereby promotes the development of type 2 cytokine‐driven eosinophilic inflammation in allergen sensitized individuals.

Although not explored in this study, modulation by LXR‐activity of the eicosanoid pathway is another potential mechanism through which LXR might exacerbate eosinophilic airway inflammation in allergen‐sensitized mice. In macrophages, LXRs have been shown to induce the expression of several genes mediating the synthesis of long‐chain polyunsaturated fatty acids, including arachidonic acid 30. Innate immune cells like macrophages may react to enrichment in arachidonic acid of membrane phospholipids by an increment in the production of eicosanoid lipid inflammatory mediators 31. Thus increased production of lipid mediators may contribute to the pro‐inflammatory function of LXR activity in allergen‐exposed mice.

In a broader context, our results indicate that LXR activity may underlie the positive association found in several epidemiological studies between elevated cholesterol levels in the blood and asthma symptom severity 4, 5, 6, 7, 8, 9, 10. Hypercholesterolemia as a result of high cholesterol diet increases the availability of oxidized cholesterol derivatives serving as LXR agonists and hence LXR transcriptional activity. It is noteworthy in this context that mice deficient in LXR activity do not show any difference in plasma cholesterol levels and accordingly are not naturally hyper‐ or hypocholesterolemic 22. Our study implies that while LXR activation promotes removal of excess cholesterol through reverse cholesterol transport, it might also be responsible for exacerbating asthma symptoms by enhancing type 2 cytokine expression from allergen‐specific T‐cells, thereby mediating increased airway inflammation similar to the inflammatory features we observed in LXR agonist‐treated WT mice. These findings are highly relevant due to the increasing prevalence of asthma in developed and developing countries where hypercholesterolemia is rising as well.

## Conflict of Interest

None declared.

## Supporting information

Additional supporting information may be found in the online version of this article at the publisher's web‐site

**Figure S1**. Gating strategy of flow cytometry‐based differential cell counting on BAL cells. Singlet cells were gated based on forward and side scatter. T cells were gated as CD3^+^ autofluorescent^−^ cells and further subdivided in CD4^+^ T cells and CD8^+^ T cells based on the respective surface markers. Subsequent NOT gates were used to identify alveolar macrophages, which were identified as CD11c^+^ autofluorescent^+^ cells, eosinophils as SiglecF^+^ CCR3^+^ cells, and neutrophils as CD11b^+^ Ly6G^+^.
