# Supplementary figures and images for: Cholesterol‐sensing liver X receptors stimulate Th2‐driven allergic eosinophilic asthma in mice
Source: Immun Inflamm Dis. 2016 Aug 2;4(3):350–61. doi: 10.1002/iid3.118 (PMC5004289; doi:10.1002/iid3.118)

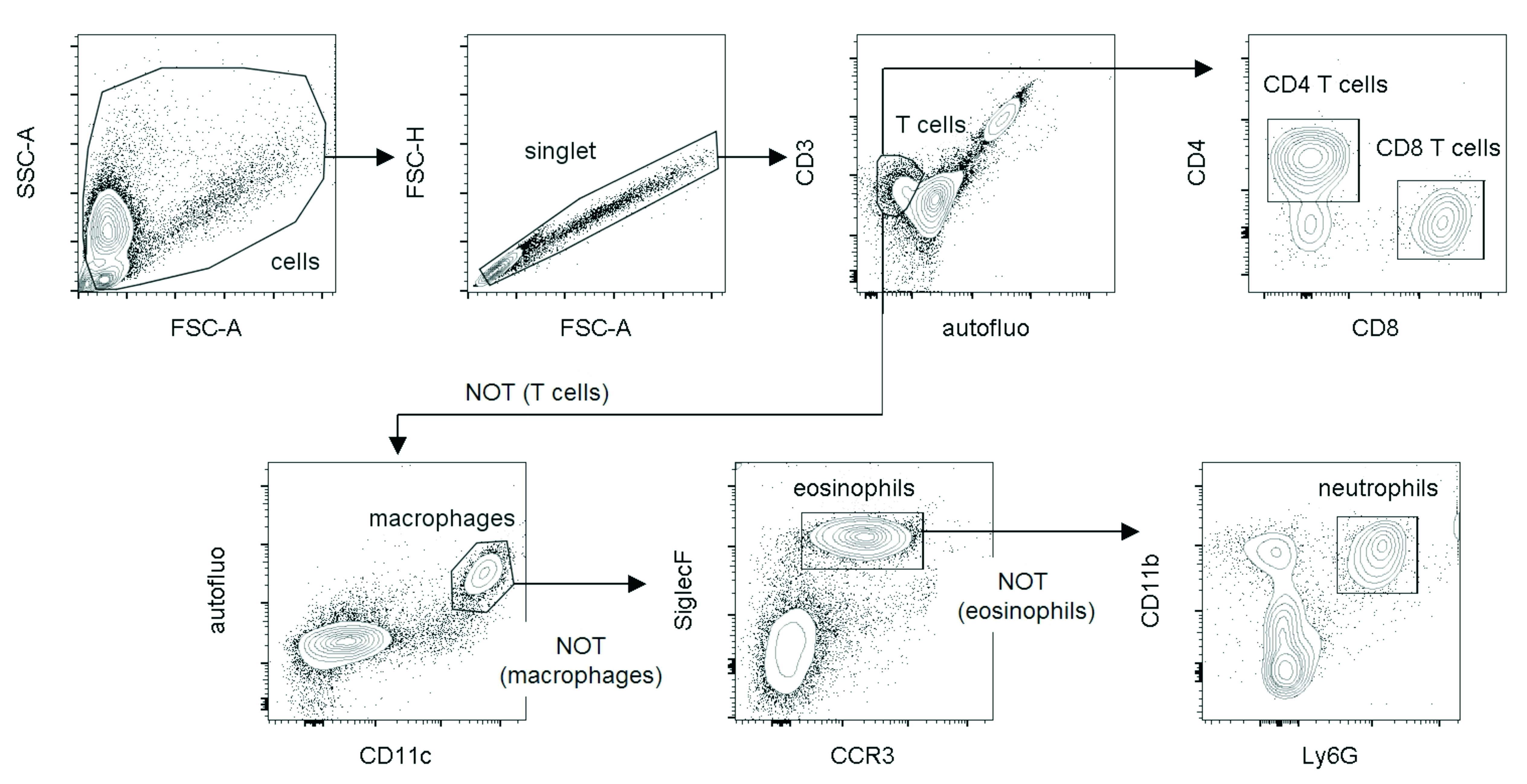

Supplement: Supplementary file 1 — Figure S1. Gating strategy of flow cytometry‐based differential cell counting on BAL cells. Singlet cells were gated based on forward and side scatter. T cells were gated as CD3+ autofluorescent− cells and further subdivided in CD4+ T cells and CD8+ T cells based on the respective surface markers. Subsequent NOT gates were used to identify alveolar macrophages, which were identified as CD11c+ autofluorescent+ cells, eosinophils as SiglecF+ CCR3+ cells, and neutrophils as CD11b+ Ly6G+. [file IID3-4-350-s001.tif]
